# Clinical impact of an anti-biofilm Hydrofiber dressing in hard-to-heal wounds previously managed with traditional antimicrobial products and systemic antibiotics

**DOI:** 10.1093/burnst/tkaa004

**Published:** 2020-03-04

**Authors:** Daniel G Metcalf, Philip G Bowler

**Affiliations:** Science & Technology, Research & Development, ConvaTec Ltd., Deeside, Flintshire, UK

**Keywords:** Antibiotics, Antimicrobials, AQUACEL Ag+ Extra, Biofilm, Dressings, Iodine, Hard-to-heal wounds, Polyhexamethylene biguanide, Silver

## Abstract

**Background:**

Hard-to-heal wounds are often compromised by the presence of biofilm. This presents an infection risk, yet traditional antimicrobial wound care products and systemic antibiotics are often used despite the uncertainty of therapeutic success and wound progression. The aim of this study was to investigate the clinical impact of a next-generation anti-biofilm Hydrofiber wound dressing (AQUACEL Ag+ Extra[AQAg+ E]) in hard-to-heal wounds that had previously been treated unsuccessfully with traditional silver-, iodine- or polyhexamethylene biguanide (PHMB)-containing dressings and products and/or systemic antibiotics.

**Methods:**

Clinical case study evaluations of the anti-biofilm dressing were conducted, where deteriorating or stagnant wounds were selected by clinicians and primary dressings were replaced by the anti-biofilm dressing for up to 4 weeks, or as deemed clinically appropriate, with monitoring via case report forms. The data was stratified for cases where traditional silver-, iodine- or PHMB-containing products, or systemic antibiotics, had been used prior to the introduction of the anti-biofilm dressing.

**Results:**

Sixty-five cases were identified for inclusion, wounds ranging in duration from 1 week to 20 years (median: 12 months). In 47 (72%) cases the wounds were stagnant, while 15 (23%) were deteriorating; 3 wounds were not recorded. After an average of 4.2 weeks of management with the anti-biofilm dressing (range: 1–11 weeks), in 11 (17%) cases the wounds had healed (i.e. complete wound closure), 40 (62%) wounds improved, 9 (14%) wounds remained the same and 5 (8%) wounds deteriorated.

**Conclusions:**

The introduction of this anti-biofilm dressing into protocols of care that had previously involved wound management with traditional antimicrobial products and/or antibiotics was shown to facilitate improvements in the healing status of most of these hard-to-heal wounds. Dressings containing proven anti-biofilm technology, in combination with antimicrobial silver and exudate management technology, appear to be an effective alternative to traditional antimicrobial products and antibiotics in the cases presented here. The use of antimicrobial wound dressings that contain anti-biofilm technology may have a key role to play in more effective wound management and antibiotic stewardship.

## Background

Infection status in chronic, hard-to-heal wounds is often uncertain, even to experienced wound care practitioners. A growing body of evidence shows that wounds that are not healing as expected, despite receiving an optimal standard of care (e.g. compression, offloading, moisture management), are compromised by biofilm [1–4]. However, treatment strategies are highly variable due to the uncertainty of infection [5] or biofilm status [6], and this often prompts the administration of systemic antibiotics as cover for a possible evolving wound infection. This is a common approach, despite uncertain therapeutic success [7] when considering the generally narrow spectrum of activity of antibiotics within a complex and diverse microflora [8], presence of biofilm and in cases with inadequate tissue perfusion [9–10]. Additionally, the presence of complex multi-species wound biofilm provides an optimal environment for exacerbation of antibiotic resistance [11]. Consequently, the likely success of systemic antibiotics in the treatment of hard-to-heal wounds should be carefully considered [12].

Treatment strategies for such wounds should undoubtedly include wound debridement and cleansing to remove unwanted materials (slough, necrosis, biofilm) prior to antimicrobial therapy [13]. Appropriate antiseptic dressings have advantages over antibiotics in that they provide broader-spectrum activity; the antimicrobial agent is active only in the local wound environment [14] and the probability of microbial resistance developing is significantly lower [15]. Consequently, antiseptic dressings should be considered as a first-line approach to wound infection management within standard care in the absence of clinical signs of spreading infection. Addressing overuse of antibiotics in wound management is imperative [14], and use of appropriate antiseptic dressings is an important consideration in antibiotic stewardship initiatives.

A next-generation anti-biofilm dressing (AQUACEL Ag+ Extra[AQAg+ E]) has been introduced, which incorporates metal-chelating and surfactant components, in an established ionic silver-containing carboxymethylcellulose (Hydrofiber) dressing [16]. The metal chelator and surfactant have demonstrated synergy with the ionic silver, resulting in disruption of biofilm structure to enable ionic silver to access and kill microorganisms within the biofilm structure [16]. A real-world clinical evaluation of AQAg+E in the management of stalled or deteriorating wounds was previously conducted [17], where various wound management methodologies and devices, including some instances of systemic and topical antimicrobial treatments, prior to introduction of AQAg+E had been recorded. In the current evaluation, data on those wounds previously managed with the most commonly-used topical antiseptic treatments, and/or systemic antibiotics, was specifically re-evaluated in more detail to better understand the success of different antimicrobial treatment strategies in the management of hard-to-heal wounds.

## Methods

Safety and effectiveness clinical evaluations of AQAg+E have previously been conducted in the United Kingdom and Ireland [17]. Deteriorating or stagnant wounds being managed with standard care protocols (e.g. compression or offloading according to wound etiology, moisture management requirements and infection risk) had been selected by clinicians, and only the primary dressing was replaced by AQAg+E for up to 4 weeks, or as deemed clinically appropriate. Patient and wound baseline and outcome data, including prior antimicrobial management strategies, were captured using detailed case report forms, as detailed previously [17]. In the present evaluation, these forms were subsequently further analysed for cases where traditional silver-, iodine- or polyhexamethylene biguanide (PHMB)-containing products, or systemic antibiotics, were used prior to the introduction of AQAg+E into otherwise standard care protocols.

Cohort baseline patient and wound data was recorded, along with clinical signs of infection, and the suspicion of wound biofilm, based on visual signs and non-visual or indirect indicators [18–19].

Wound outcomes were defined as wound deterioration, stagnation, improvement (clinician’s subjective opinion) or healing (defined as full re-epithelialisation with no volume). Wound exudate levels, wound bed appearance, in terms of approximate tissue type coverage, and skin health were recorded.

## Results

### Baseline

Patient and wound baseline data for the 65 cases included in this evaluation is shown in Table 1. The most frequently adopted antimicrobial wound management strategies before the introduction of AQAg+E were single-mode approaches: standard silver dressings alone (26%), followed by iodine dressings alone (23%), antibiotics alone (12%) and then PHMB products alone (11%). Combinations thereof were also observed, the most frequent being silver dressings with antibiotics (9%) and silver dressings with PHMB products (6%). The 65 wounds ranged in duration from 1 week to 20 years (median: 12 months). In 47 (72%) cases, the wounds were stagnant, while 15 (23%) were deteriorating; 3 wounds were not recorded.

**Table 1:** Patient and wound baseline data for patients with wounds previously being managed with protocols including silver-, iodine-, or polyhexamethylene biguanide-containing wound dressings or products, and/or systemic antibiotics

Characteristic	Number
Number of patients with wounds	65
Patient sex (F/M)	35/30
Patient age (years)	Mean 69(Range 18–90); Median 74
Wound durations (months)	Mean 32(Range 0.25–240); Median 12 (7 not recorded)
Wound status	47 stagnant; 15 deteriorating (3 not recorded)
Wound infection status	18 infected; 29 not infected (18 not recorded)
Wound types	
Venous	17
Mixed	10
Pressure ulcer	6
Arterial	5
Lymphovenous	3
Leg ulcer	3
Surgical/post-op	3
Diabetic foot ulcer	2
Cyst	2
Graft	2
Varicose eczema	1
Trauma	1
Pilonidal sinus	1
Scalp	1
Pilonidal sinus excision	1
Donor site	1
Amputation	1
Not recorded	5
Wound locations	
Leg	38
Foot	11
Abdomen	2
Ankle	1
Axilla	1
Buttock cleft	1
Hip	1
Leg & abdomen	1
Malleolus	1
Pilonidal sinus	1
Sacrum	1
Scalp	1
Sternum	1
Thigh	1
Toe/amputation	1
Not recorded	2
Previous antimicrobial type used	
Standard silver dressings	17
Iodine dressings	15
PHMB products	7
Silver & iodine dressings	2
Silver, iodine & PHMB products	1
Silver & PHMB products	4
Iodine & PHMB products	2
Systemic antibiotics	8
Silver dressings & antibiotics	6
Iodine dressings & antibiotics	1
PHMB dressings & antibiotics	1
Silver, PHMB dressings & antibiotics	1
Frequency of dressing change	
Daily	10
Every 2 days	13
Twice weekly	1
Every 3 days	22
Every 4 days	5
Weekly	4
Not recorded	10

*PHMB* polyhexamethylene biguanide, *F* female, *M* male.

**Figure 1.**
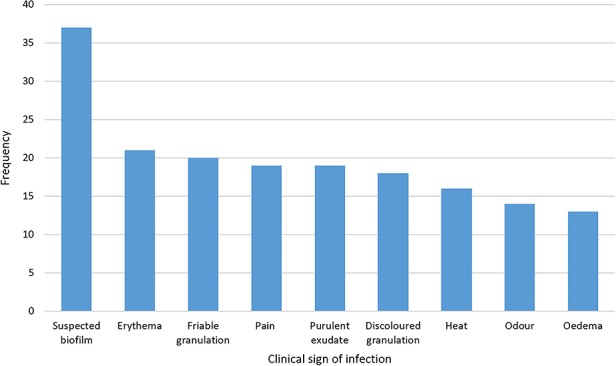
Frequency with which clinical signs associated with wound infection were reported (*n* = 65)

The most frequently reported clinical sign associated with wound infection was biofilm suspicion (Fig 1) (*n* = 37; 57% of cases), based on visual and indirect indicators of biofilm [18–19].

### Outcomes

**Figure 2.**
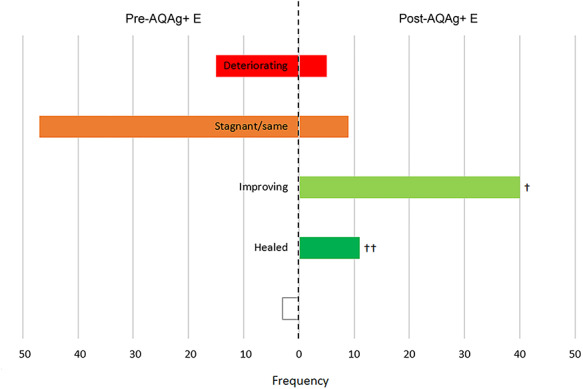
Wound statuses before and after the introduction of AQUACEL Ag+ Extra (AQAg+ E) dressing (*n* = 65). *Open box* not recorded, † 1 patient remained on systemic antibiotics concurrently with AQAg+ E, †† 2 patients remained on systemic antibiotics concurrently with AQAg+ E

After an average of 4.2 weeks of wound management with AQAg+E (range: 1–11 weeks), in 11 (17%) cases the wounds had healed (full re-epithelialisation with no volume), 40 (62%) wounds improved, 9 (14%) wounds remained the same and 5 (8%) wounds deteriorated (Fig 2). It should be noted that in 2 of the wounds that healed, and in one that improved, antibiotics were continued concurrently following the introduction of AQAg+E.

Each of the 5 wounds that were classed as deteriorating at the end of the AQAg+E evaluation period had previously been managed unsuccessfully with 2 different types of antimicrobial agent: silver dressings had been combined with topical steroid/antibiotic cream (2 wounds on same patient with lymphovenous foot and leg ulcers, who was eventually prescribed systemic antibiotics), systemic antibiotics (an axillary cyst and a varicose eczema leg wound) or PHMB gel (an arterial leg ulcer). These complex wounds ranged in duration from 10 to 84 months, and 4 displayed 3–5 clinical signs of infection.

**Figure 3.**
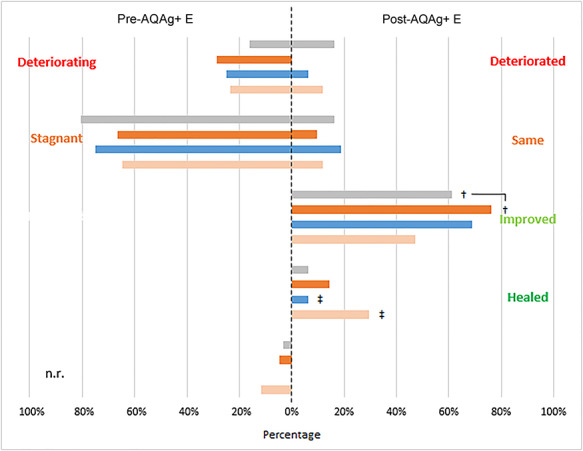
Wound statuses before and after the introduction of AQUACEL Ag+ Extra (AQAg+ E) dressing. (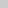) protocols including standard silver dressings (*n* = 31), (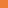) protocols including iodine dressings (*n* = 21), (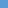) protocols including polyhexamethylene biguanide (PHMB) products *(n* = 16); (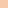) protocols including antibiotics (*n* = 17), † 1 patient was previously being managed with both silver and iodine dressings, ‡ these 2 patients remained on systemic antibiotics concurrently with AQAg+ E. *n.r.* not recorded

Wound outcomes prior to, and after introduction of AQAg+E, analysed by prior antimicrobial therapy are shown in Fig 3. This shows that wounds statuses before the switch to AQAg+E were generally stagnant or deteriorating, irrespective of antimicrobial therapy. Following the introduction of AQAg+E, the wounds with the highest full healing response were those managed previously with antibiotics, where 29% (*n* = 5) of wounds went on to fully heal after AQAg+E introduction (one of these patients remained on antibiotics concurrently with AQAg+E), compared to, for example, 6% (*n* = 2) of those wounds previously managed with silver dressings.

**Figure 4.**
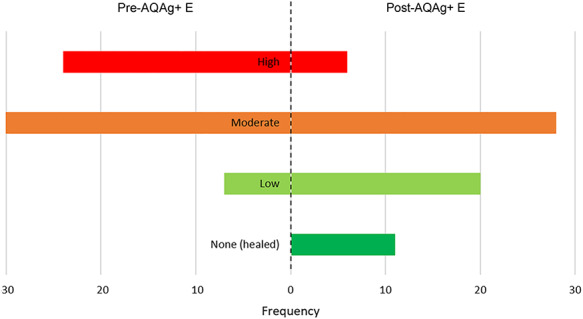
Exudate levels of wound being managed before and after the introduction of AQUACEL Ag+ Extra (AQAg+ E) dressing (*n* = 65)

Exudate levels of wounds being managed prior to the introduction of AQAg+E were mainly moderate (52%; *n* = 24) or high (37%; *n* = 34) (Fig 4), which changed to mainly low (31%; *n* = 20) or moderate (43%; *n* = 28) following the introduction of AQAg+E. Only 9% (*n* = 6) of wounds had high levels of exudate after the switch, while the 11 healed wounds produced no exudate.

**Figure 5.**
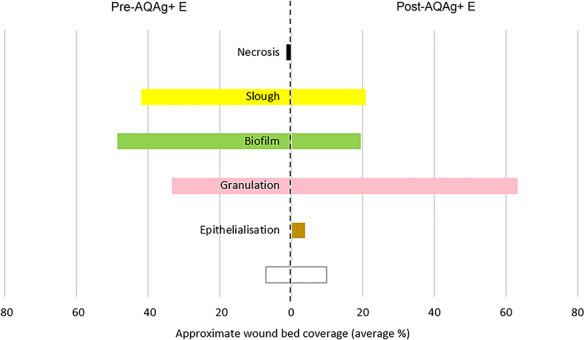
Wound bed tissue types before and after the introduction of AQUACEL Ag+ Extra (AQAg+ E) dressing (*n* = 65). *Open box* not recorded

Approximate wound bed tissue classifications before the introduction of AQAg+E were mainly 49% suspected biofilm [18–19] and 42% suspected slough (Fig 5). Following the introduction of AQAg+E, this changed to mainly 63% granulation tissue. The total ‘unwanted’ wound bed tissues (necrosis, slough, biofilm) reduced from 92% to 40% following the introduction of AQAg+E, while the total ‘healthy’ wound bed tissues (granulation and epithelial tissue) increased from 33% to 67%.

Table 2 shows that the peri-wound skin health which was largely dry/eczematous (44% of those recorded) or macerated (24%) before the introduction of AQAg+E, and was mainly improved (67% of those recorded) following the switch to AQAg+E.

## Discussion

Chronic and acute wounds impeded by microbial biofilm present a significant challenge to the effectiveness of both topical antiseptics and systemic antibiotics, and hence wound progression. Biofilm-based wound care involving multimodal approaches to controlling wound debridement (e.g. sharp or soft debridement, cleansing and topical antimicrobial agents) is increasingly acknowledged as an important strategy in the management of hard-to-heal wounds [20–21]. Although the requirement for combination anti-biofilm/antimicrobial technologies has been acknowledged [11], few are currently designed and available to break down biofilm and maximise the effectiveness of topical antiseptics (and, potentially, systemic antibiotics).

AQAg+E is a novel dressing technology that has been designed specifically for this purpose [16]. The synergistic combination of a metal chelator, surfactant and antimicrobial agent has been demonstrated *in vitro* [16,22–24] and *in vivo* [25] to combat biofilm and facilitate wound healing [26]. The current analysis was undertaken to review the effectiveness of systemic antibiotics and topical antimicrobial agents within protocols used to manage hard-to-heal wounds, and then to compare this with outcomes in the same group of wounds when primary dressings were replaced with AQAg+E. Considering that all of the wounds analysed were either deteriorating or stalled, the use of systemic antibiotics and silver-, iodine- and PHMB-containing products clearly had minimal impact on wound progression. This perhaps emphasises the likely involvement of biofilm in these hard-to-heal wounds, and its tolerance to standard antimicrobial therapies.

**Table 2:** Skin health for patients with wounds previously being managed with protocols including silver-, iodine-, or polyhexamethylene biguanide-containing wound dressings or products, and/or systemic antibiotics, and then after the introduction of AQUACEL Ag+ Extra dressing (*n* = 65)

Skin assessment	Number
Skin health after management with protocolsincluding standard antimicrobials	
Healthy	10
Part healthy, part macerated	1
Dry-eczematous	24
Macerated	13
Part macerated, part dry-eczematous	5
Fragile	1
Not recorded	11
Change in skin after switching to protocols including AQUACEL Ag + Extra dressing	
Improved	40
Same	16
Deteriorated	4
Not recorded	5

When AQAg+E, a dressing designed to combat wound biofilm, was introduced into management protocols, 79% of the wounds either healed completely (fully re-epithelialised) or improved. Although we do not have direct evidence for the mode of action of the dressing in these cases, the significant improvement in wound progression leads us to hypothesise that the anti-biofilm technology of the dressing was effective in disrupting wound biofilm and enabling the antimicrobial silver component of the dressing to work more effectively, where, previously, standard antimicrobial agents had been less effective, as has been observed *in vitro* [16,22–24] and *in vivo* [25]. Despite the reported anti-biofilm effectiveness of some silver-, iodine- and PHMB-containing products *in vitro* [27–29], *in vivo* [30–32] and in clinical studies [33–34], in the cases presented in this study, such standard antimicrobial agents were not supporting wound progression.

The small subset of wounds that were deteriorating after the introduction of AQAg+E were complex, longstanding wounds, some of which involved spreading infection. This could explain their lack of progress following the introduction of the new anti-biofilm technology. Complex comorbidities or systemic factors are unlikely to be addressed effectively by a change in topical antimicrobial dressing alone.

Limitations of this small clinical evaluation include the fact that wound outcomes (except for healed wounds), exudate levels and wound bed appearance were based on the subjective opinions of clinicians, which reflects the current lack of widely available clinical tools for standardised assessment of many of these wound characteristics. In future dressing evaluations of this type, laboratory techniques such as confocal laser scanning microscopy and electron microscopy [35] could be used to establish whether samples from non-healing wounds contained biofilm. Wound size and time-to-healing data was not available in all the wounds included in the evaluation, so was excluded from the aggregate data analysis. Although the evaluations were designed so that standard of care was conducted throughout, with the only change in wound management being the switch of primary dressings from the incumbent to AQAg+E, the exact care details were not recorded for every patient, so the assumption was made that the standard of care was maintained throughout.

## Conclusions

The results from this small clinical case analysis emphasise the importance of dressings and dressing technology in encouraging healing in previously hard-to-heal wounds. Because of the uncertainty of infection status in chronic wounds with non-obvious signs of infection, systemic antibiotics are frequently and overly used in wound management, and often with poor clinical outcomes. Biofilm is one reason why systemic antibiotic therapy is often unsuccessful in these challenging wounds. Consequently, new strategies are required that are able to combat wound biofilm. AQAg+E wound dressing has been designed with anti-biofilm technology to facilitate biofilm control in challenging wounds, and evidence from this small clinical analysis indicates its clinical value. Larger, controlled clinical studies that also establish biofilm presence are warranted to investigate these observations further. This study emphasises that the use of appropriate antimicrobial dressing technologies can play a significant role in the successful management of hard-to-heal wounds, and also potentially reduce the need for systemic antibiotic therapy, thereby assisting in antimicrobial stewardship initiatives in wound care.

## Data Availability

The datasets used and/or analysed during the current study are available from the corresponding author on reasonable request.

## Abbreviations

AQAg+E, AQUACEL Ag+ Extra; PHMB, polyhexamethylene biguanide.

## References

[1] Metcalf DG, Bowler PG Biofilm delays wound healing: A review of the evidence. Burns Trauma 2013;1:5–12. 27574616 10.4103/2321-3868.113329 PMC4994495

[2] Malone M, Bjarnsholt T, McBain AJ, James GA, Stoodley P, Leaper D, et al. The prevalence of biofilms in chronic wounds: A systematic review and meta-analysis of published data. J Wound Care 2017;26:20–5. 28103163 10.12968/jowc.2017.26.1.20

[3] Wolcott RD Biofilms cause chronic infections. J Wound Care 2017;26:423–5. 28795886 10.12968/jowc.2017.26.8.423

[4] Webb R A chronic case of confusion. J Wound Care 2017;26:421. 28795883 10.12968/jowc.2017.26.8.421

[5] Reddy M, Gill SS, Wu W, Kalkar SR, Rochon PA Does this patient have an infection of a chronic wound? JAMA 2012;307:605–11. 22318282 10.1001/jama.2012.98

[6] Haesler E, Swanson T, Ousey K, Carville K Clinical indicators of wound infection and biofilm: reaching international consensus. J Wound Care 2019;28:s4–12. 10.12968/jowc.2019.28.Sup3b.S4 30840533

[7] Lipsky BA, Dryden M, Gottrup F, Nathwani D, Seaton RA, Stryja J Antimicrobial stewardship in wound care: A position paper from the British Society for Antimicrobial Therapy and the European wound management association. J Antimicrob Chemother 2016;71:3026–35. 27494918 10.1093/jac/dkw287

[8] Bowler PG, Duerden BI, Armstrong DG Wound microbiology and associated approaches to wound management. Clin Microbiol Rev 2001;14:244–69. 11292638 10.1128/CMR.14.2.244-269.2001 PMC88973

[9] Pereira SG, Moura J, Carvalho E, Empadinhas N Microbiota of chronic diabetic wounds: Ecology, impact and potential for innovative treatment strategies. Frontiers Microbiol 2017;8:1791. 10.3389/fmicb.2017.01791 PMC5613173 28983285

[10] Vella J, Vella M, Cassar K, Camilleri L, Serracino-Inglott A, Azzopardi LM, et al. Factors affecting penetration of ciprofloxacin in lower extremity ischemic tissues. Int J Low Extrem Wounds 2016;15:126–31. 26711367 10.1177/1534734615623707

[11] Bowler PG Antibiotic resistance and biofilm tolerance: A combined threat in the treatment of chronic infections. J Wound Care 2018;27:273–7. 29738295 10.12968/jowc.2018.27.5.273

[12] Rahim K, Saleha S, Zhu X, Huo L, Basit A, Franco OL Bacterial contribution in chronicity of wounds. Microb Ecol 2017;73:710–21. 27742997 10.1007/s00248-016-0867-9

[13] Wolcott RD, Kennedy JP, Dowd SE Regular debridement is the main tool for maintaining a healthy wound bed in most chronic wounds. J Wound Care 2009;18:54–6. 19418781 10.12968/jowc.2009.18.2.38743

[14] Roberts CD, Leaper DJ, Assadian O The role of topical antiseptic agents within antimicrobial stewardship strategies for prevention and treatment of surgical site and chronic open wound infection. Adv Wound Care (New Rochelle) 2017;6:63–71. 28224049 10.1089/wound.2016.0701 PMC5286547

[15] McDonnell G, Russell AD Antiseptics and disinfectants: Activity, action, and resistance. Clin Microbiol Rev 1999;12:147–79. 9880479 10.1128/cmr.12.1.147 PMC88911

[16] Bowler PG, Parsons D Combatting wound biofilm and recalcitrance with a novel anti-biofilm Hydrofiber wound dressing. Wound Medicine 2016;14:6–11.

[17] Metcalf D, Parsons D, Bowler P Safety and effectiveness of a new antimicrobial wound dressing designed to manage exudate, infection and biofilm. Int Wound J 2017;14:203–13. 27004423 10.1111/iwj.12590 PMC7949869

[18] Hall-Stoodley L, Stoodley P, Kathju S, Høiby N, Moser C, Costerton JW, et al. Towards diagnostic guidelines for biofilm-associated infections. FEMS Immunol Med Microbiol 2012;65:127–45. 22469292 10.1111/j.1574-695X.2012.00968.x

[19] Metcalf DG, Bowler PG, Hurlow J, A clinical algorithm for wound biofilm identification. J Wound Care 2014;23:137–43. 24633059 10.12968/jowc.2014.23.3.137

[20] Wolcott RD, Rhoads DD A study of biofilm-based wound management in subjects with critical limb ischaemia. J Wound Care 2008;17:145–55. 18494432 10.12968/jowc.2008.17.4.28835

[21] Attinger C, Wolcott R Clinically addressing biofilm in chronic wounds. Adv Wound Care (New Rochelle) 2012;1:127–32. 24527292 10.1089/wound.2011.0333 PMC3839004

[22] Said J, Walker M, Parsons D, Stapleton P, Beezer AE, Gaisford S An in vitro test of the efficacy of an anti-biofilm wound dressing. Int J Pharm 2014;474:177–81. 25151435 10.1016/j.ijpharm.2014.08.034

[23] Next-generation antimicrobial dressings: AQUACEL™ Ag+ Extra™ and Ribbon *.* London: Wounds International, 2014 (Suppl). Available to download from: www.woundsinternational.com.

[24] Parsons D, Meredith K, Rowlands VJ, Short D, Metcalf DG, Bowler PG Enhanced performance and mode of action of a novel antibiofilm Hydrofiber® wound dressing. Biomed Res Int 2016;December:7616471. 10.1155/2016/7616471 PMC5136405 27990437

[25] Seth AK, Zhong A, Nguyen KT, Hong SJ, Leung KP, Galiano RD, et al. Impact of a novel, antimicrobial dressing on in vivo, Pseudomonas aeruginosa wound biofilm: Quantitative comparative analysis using a rabbit ear model. Wound Repair Regen 2014;22:712–9. 25230854 10.1111/wrr.12232

[26] Davis SC, Li J, Gil J, Valdes J, Solis M, Higa A, et al. The wound-healing effects of a next-generation anti-biofilm silver Hydrofiber wound dressing on deep partial-thickness wounds using a porcine model. Int Wound J 2018;15:834–9. 29893025 10.1111/iwj.12935 PMC7949536

[27] Percival SL, Bowler P, Woods EJ Assessing the effect of an antimicrobial wound dressing on biofilms. Wound Repair Regen 2008;16:52–7. 18211579 10.1111/j.1524-475X.2007.00350.x

[28] Phillips PL, Yang Q, Davis S, Sampson EM, Azeke JI, Hamad A, et al. Antimicrobial dressing efficacy against mature Pseudomonas aeruginosa biofilm on porcine skin explants. Int Wound J 2015;12:469–83. 24028432 10.1111/iwj.12142 PMC7950379

[29] Kirker KR, Fisher ST, James GA, McGhee D, Shah CB Efficacy of polyhexamethylene biguanide-containing antimicrobial foam dressing against MRSA relative to standard foam dressing. Wounds 2009;21:229–33. 25903814

[30] Davis SC, Li J, Gil J, Head C, Valdes J, Glinos GD, et al. Preclinical evaluation of a novel silver gelling fiber dressing on *Pseudomonas aeruginosa* in a porcine wound infection model. Wound Repair Regen 2019;27:360–5. 30920083 10.1111/wrr.12718

[31] Roche ED, Woodmansey EJ, Yang Q, Gibson DJ, Zhang H, Schultz GS Cadexomer iodine effectively reduces bacterial biofilm in porcine wounds ex vivo and in vivo. Int Wound J 2019;16:674–83. 30868761 10.1111/iwj.13080 PMC6850490

[32] Davis SC, Harding A, Gil J, Parajon F, Valdes J, Solis M, et al. Effectiveness of a polyhexanide irrigation solution on methicillin-resistant *Staphylococcus aureus* biofilms in a porcine wound model. Int Wound J 2017;14:937–44. 28266133 10.1111/iwj.12734 PMC7950165

[33] Malone M, Johani K, Jensen SO, Gosbell IB, Dickson HG, McLennan S, et al. Effect of cadexomer iodine on the microbial load and diversity of chronic non-healing diabetic foot ulcers complicated by biofilm in vivo. J Antimicrob Chemother 2017;72:2093–101. 28402558 10.1093/jac/dkx099 PMC5890712

[34] Lenselink E, Andriessen A A cohort study on the efficacy of a polyhexanide-containing biocellulose dressing in the treatment of biofilms in wounds. J Wound Care 2011;20:534–9. 22240848 10.12968/jowc.2011.20.11.534

[35] Hurlow J, Blanz E, Gaddy JA Clinical investigation of biofilm in non-healing wounds by high resolution microscopy techniques. J Wound Care 2016;25:S11–22. 10.12968/jowc.2016.25.Sup9.S11 PMC5058422 27608736

